# Life Table Study of *Liriomyza trifolii* and Its Contribution to Thermotolerance: Responding to Long-Term Selection Pressure for Abamectin Resistance

**DOI:** 10.3390/insects15060462

**Published:** 2024-06-20

**Authors:** Yucheng Wang, Yawen Chang, Weirong Gong, Yuzhou Du

**Affiliations:** 1 College of Plant Protection, Yangzhou University, Yangzhou 225000, China; wangycyzu@163.com (Y.W.); changyawen@yzu.edu.cn (Y.C.); 2 Plant Protection and Quarantine Station of Jiangsu Province, Nanjing 210036, China; wong272827@gmail.com; 3 Joint International Research Laboratory of Agriculture and Agri-Product Safety, the Ministry of Education, Yangzhou University, Yangzhou 225000, China

**Keywords:** *Liriomyza trifolii*, abamectin resistance, *Hsps*, thermotolerance

## Abstract

**Simple Summary:**

*Liriomyza trifolii* is an invasive, highly devastating pest of horticultural and vegetable plants that causes significant outbreaks due to its thermotolerance and insecticide resistance. This study investigated the impact of long-term abamectin resistance on *L. trifolii*’s population dynamics and thermal tolerance. We compared abamectin-resistant (AB-R) and susceptible (S) strains using various parameters, including life table analysis, thermal preference, critical thermal maximum, heat knockdown times, eclosion and survival rates, and heat shock protein expression. Although long-term selection for abamectin resistance was detrimental to survival and reproduction, it activated self-defense mechanisms and rapid adaptive adjustments and conferred modest thermal tolerance, which suggests a dual nature of insecticide effects. These findings underscore the importance of considering temperature in insecticide application in the context of global climate change and provide insights into adaptive evolution to multiple environmental pressures.

**Abstract:**

*Liriomyza trifolii* is a significant invasive pest that targets horticultural and vegetable crops, causing large-scale outbreaks characterized by pronounced thermotolerance and insecticide resistance. This study examined the impact of long-term selection for abamectin resistance during the larval stage of *L. trifolii* on its population dynamics and thermal tolerance. We conducted a comprehensive comparison between the abamectin-resistant strain (AB-R) and the susceptible strain (S), including age-stage, two-sex life table analysis, thermal preference (T_pref_), critical thermal maximum (CT_max_), heat knockdown times (HKDTs), eclosion and survival rates, and *LtHsp* expression under heat stress. Our results showed that while selection for abamectin resistance was detrimental to survival and reproduction, it activated self-defense mechanisms and rapid adaptive adjustments and conferred modest thermal tolerance, which suggests a dual nature of insecticide effects. The AB-R strain exhibited significantly higher thermal preference and CT_max_ values, along with a longer HKDT and improved survival. Additionally, there was a significant upregulation of *LtHsp* expression in the AB-R strain compared to the S strain. These findings indicate that the evolution of thermal adaptation was accompanied by abamectin resistance development, emphasizing the necessity of considering temperature effects when applying chemical control. Our study provides valuable insights into how physiological acclimation may help mitigate the toxic effects of insecticides and illustrate how insects respond to multiple environmental pressures.

## 1. Introduction

*Liriomyza trifolii* is a significant invasive pest that targets horticultural and vegetable crops [1,2]. Female adults inflict damage by piercing leaves with their ovipositor to lay eggs, with both females and males feeding on plant nutrients through these wounds [3,4]. These puncture wounds also serve as entry points for the transmission of plant viruses and fungal diseases, causing extensive plant cell necrosis [3]. The larvae hatched from these eggs tunnel through mesophyll tissue, creating twisting paths and excreting harmful substances [4,5], which are detrimental to photosynthetic capacity, leaf respiration, and substance transport, ultimately resulting in leaf withering, necrosis, abscission, and seedling death [6]. Once mature, larvae bite through the upper epidermis of leaves, climb out of the tunnels, pupate, and continue their destructive cycle upon emergence [1,7]. Since the outbreak of *Liriomyza* species in the last century and their subsequent spread to various parts of the world, chemical control remains the primary management strategy globally, with abamectin and its derivatives being the most extensively utilized due to their broad-spectrum efficacy [8,9]. Abamectin, a highly effective neurotoxin, exhibits broad-spectrum insecticidal, acaricidal, and nematocidal properties [10,11], and it can also damage antioxidant systems, leading to oxidative stress, apoptosis, and the inhibition of autophagy [11,12,13]. Unfortunately, the excessive and frequent use of such pesticides has led to the significant development of resistance, further endangering crop productivity [8,14].

*L. trifolii* spreads rapidly, particularly during hot weather, and is characterized by its robust thermotolerance and insecticide resistance [15,16]. Adaptability and tolerance to adverse environments play a crucial role in the invasiveness and dissemination of *L. trifolii* [16,17]. In response to different stresses, insects undergo self-optimization and adaptation, affecting various processes, such as metabolic rate, feeding, digestion, growth, assimilation, and development [18,19]. Previously, we developed a strain of *L. trifolii* resistant to abamectin (AB-R) [20], demonstrating that the species was prone to develop cross-resistance to other insecticides with similar ingredients, such as a 0.2% *w*/*w* microemulsion of abamectin + 19.8% monosultap (also known as ‘Banqianjing’). This targeted process of domestication may result in insects focusing on the development of a specific adaptive response, but this always comes with a fitness cost [21]. The mechanisms by which insects establish trade-offs between gains and losses and optimize themselves in adverse conditions, as well as the consequences of enhanced insecticide resistance for their life history and performance, remain poorly understood.

Insect tolerance to various environmental stressors often involves multiple mechanisms [22,23]. In the context of global warming, the negative effects of pesticides on insects might be reduced through thermal adaptation and increased degradation rates [24,25,26]. Our previous research supports the argument that *L. trifolii* displays adaptive cross-tolerance to insecticides and elevated temperatures, and the evolution of thermal adaption coincides with enhanced insecticide tolerance [20]. Heat shock proteins (HSPs) are stress-responsive proteins in insects that safeguard host proteins from diverse stressors, and their expression is modulated by thermal changes [27,28]. The elevated production of HSPs indicates an insect’s tolerance to high temperatures [15]. Our earlier findings indicated that the overuse of insecticides could potentially lead to the adaptive evolution of HSP-mediated thermotolerance in *L. trifolii*, demonstrating that thermal adaption in *L. trifolii* coincided with the emergence of abamectin resistance. Therefore, a further comprehensive assessment of these synergistic effects can provide valuable insights into the evolutionary trajectory of populations, which can increase the precision of predicting the effects of climate change on pests [29].

To investigate the potential impact of thermal and insecticide tolerance on the reproductive success of *L. trifolii* and its evolutionary trajectory, we conducted a thorough comparative analysis between an abamectin-resistant strain and a susceptible strain, encompassing life table parameters, preferred (T_pref_) and critical thermal maximum (CT_max_) temperatures, heat knockdown times (HKDTs), the eclosion rates of pupae, the survival rates of adults, and the expression of heat shock proteins (*LtHsps*) under heat stress. These results will not only improve our comprehension of the pest’s self-optimization and adaptation mechanisms under multiple environmental pressures but also assist in developing more effective pest monitoring and management strategies, which is crucial in the context of global climate change and agricultural sustainability.

## 2. Materials and Methods

### 2.1. Insects

*L. trifolii* was initially gathered from celery (*Apium graveolens*) in Yangzhou (32.39° N, 119.42° E) and maintained only on kidney beans (*Phaseolus vulgaris*) at 25 ± 1 °C with a 16:8 light/dark photoperiod for nearly a decade without insecticide exposure (designated the S strain), as described by Chen and Kang [30]. Leaves exhibiting tunnels were gathered for pupation, and newly emerged adults were released into cages with fresh kidney beans for mating and egg laying. The cages were rectangular frames (35 × 35 × 50 cm) and were covered with fine nylon mesh netting to ensure proper ventilation and prevent the insects from escaping.

The abamectin-resistant derivative of *L. trifolii* (AB-R strain) used in this study was derived from the S strain, and the resistance ratio was 33.912-fold, as described previously [20]. The selection process for resistance was accomplished using 3% abamectin EC (Anhui Sida Pesticide Chemical Co., Ltd., Hefei, China). A leaf-dip method was developed to assay insecticide toxicity in *L. trifolii* larvae, as described before [20], and DPS version 9.01 software (DPS, Hangzhou, China) was used to calculate the median lethal concentrations (LC_50_) with 95% confidence intervals (CIs). Bean leaves infested by 3-day-old larvae of *L. trifolii* were treated daily with the LC_50_ dosage of abamectin until pupation.

### 2.2. Determination of Physiological Parameters

In the life table analysis, 15 pairs of newly emerged *L. trifolii* males and females were introduced into rearing cages with 40 fresh bean seedlings for mating and oviposition. After 12 h, the eggs in the leaves were counted under a microscope (EZ4, Leica, Wetzlar, Germany). The viable density of eggs was maintained at five per leaf, and any excess eggs were punctured with a small needle to prevent overviability, ensuring a consistent density. The development and survival rates of eggs and larvae were meticulously documented on a daily basis. Every 10 pupae (3 days old) were weighed using a high-precision electronic balance (BSA124S-CW, Sartorius, Göttingen, Germany), and the mean weights were calculated. The length and width of each 3-day-old pupa were measured and photographed through a microscope (S9i, Leica, Wetzlar, Germany) and its imaging system with a built-in scale plate (Leica Application Suite v. 4.12.0, Leica, Wetzlar, Germany). The emergence rate and development of pupae were recorded daily.

Upon emergence, a single pair of adults was placed into a 700 mL transparent plastic cup (9 × 18 cm) containing a fresh kidney bean seedling. Each strain included 15 replicates, and the surviving adults were moved to a new bean seedling daily. Fecundity and survival rates were recorded every day until their eventual demise.

### 2.3. Evaluation of Heat Knockdown Behavior

The thermotolerance of *L. trifolii* was investigated by determining the critical thermal maximum (CT_max_) values and heat knockdown times (HKDTs) by observing activity under elevated temperatures. The knockdown assessment of individuals was conducted in 24-well plexiglass plates placed in a customized incubator (JM-CXG-32, Jiangsu Jiamei Instrument Manufacturing Co., Ltd., Changzhou, China) equipped with a monitoring camera and a sensor for real-time temperature recording. Knockdown was declared when adult leafminers dropped from the interior walls to the well bottom and were unable to climb back up, irrespective of movement [20]. Each strain included 30 pairs of adults, with individuals showing no discernible behaviors being excluded from the analysis.

The methodology used to measure CT_max_ values in *L. trifolii* followed established protocols [31,32]. Newly emerged adults were arranged in separate wells of a 24-well microtiter plate, each with tiny holes in the top for air circulation. The plates were subsequently moved into an incubator and exposed to a starting temperature of 25 °C with incremental increases of 0.2 °C per minute until reaching 50 °C.

HKDT values were assessed using the standardized protocols described by Nyamukondiwa and Terblanche [33]. The time (in hours) recorded for an adult insect to lose its self-righting ability was referred to as the HKDT [34]. Treatment temperatures for HKDT assessment were selected in accordance with the CT_max_ data.

### 2.4. Measurement of Thermal Preference

The preferred temperature (T_pref_) of *L. trifolii* was determined by exposing adults to a thermal gradient encompassing various surface temperatures and tracking their movements over time [35,36,37]. A linear thermal gradient was established using an aluminum plate (50 × 6 × 5.5 cm), where surface temperatures varied from 17 °C to 32 °C, with one end immersed in a water bath circulator at 60 °C (DC-3010, Ningbo Scientz Biotechnology Co., Ltd., Ningbo, China) and the other end embedded in dry ice. The aluminum plate had six lanes and was covered with transparent acrylic panels with small holes. After the temperature stabilized, a newly emerged adult was placed in the middle of a lane. The temperature of the adult’s position was measured every ten minutes; abnormally excited adults that crawled constantly were excluded.

### 2.5. Temperature Treatments and Expression of LtHsps

To determine the *LtHsp* expression of different *L. trifolii* strains in response to thermal stress, we collected newly emerged adults (*n* = 20) and exposed them to temperatures of 40, 42.5, and 45 °C for 0.5, 1, 1.5, and 2 h using a temperature controller (DC-3010, Ningbo Scientz Biotechnology Co., Ltd., Ningbo, China); control insects were treated at 25 °C. Each treatment was conducted in three biological replicates.

Total RNA was extracted from *L. trifolii* using RNA-easy reagent (#R701, Vazyme, Nanjing, China). First-strand cDNA was synthesized from RNA using the HiScript II Q RT SuperMix for qPCR (+gDNA wiper) Kit (#R223, Vazyme, China). Quantitative real-time PCR (qRT-PCR) was performed with the CFX-96 Real-Time PCR System (Bio-Rad, Berkeley, CA, USA) using primers designed with Premier 5.0 (Table S1). Reactions were carried out in 20 μL volumes containing 1 μL of each gene-specific primer (10 μM), 10 μL of ChamQ QSYBR qPCR Master Mix (2×) (#Q311, Vazyme, China), 2 μL of cDNA (diluted tenfold), and 6 μL of ddH_2_O. *Actin* was utilized as an internal reference gene, and relative mRNA levels were calculated using the 2^−ΔΔCt^ method [38,39]. Each treatment had four replicates, and each reaction was assessed in triplicate.

### 2.6. Statistical Analysis

The raw data were analyzed using the age-stage, two-sex life table method and the TWOSEX-MSChart program designed in Visual BASIC 6 (Service Pack 4) (Taiwan, China) and are accessible at http://140.120.197.173/Ecology/prod02.htm (accessed on 30 March 2024) [40,41,42,43]. Taking into account the initial sex ratio and more data on immature individuals, the bootstrap matching technique was employed to combine the immature data (e.g., survival and duration of each stage) and the adult data (e.g., adult longevity and daily fecundity of females) for the construction of a complete life table [44]. After basic analysis, the bootstrap technique was used with 10,000 resamples to estimate the standard errors of the population parameters [45,46,47,48,49]. Differences between the two strains were evaluated by the paired bootstrap test on the 95% confidence interval and t-intervals of 100,000 differences [50,51,52].

The age-stage-specific survival rate (*s_xj_*) represents the probability that a newly laid egg will survive to age *x* and stage *j* and is calculated as $s_{xj}=\frac{n_{xj}}{n_{01}}$, where *n_xj_* is the number of surviving individuals at age *x* and stage *j*, and *n*_01_ is the number of eggs used for the life table analysis [53]. The age-specific survival rate (*l_x_*) is the possibility that a newborn survives to age *x* and is calculated as $l_{x}=\sum_{j=1}^{\beta }s_{xj}$, where β represents the number of life stages. The age-stage-specific fecundity (*f_xj_*) gives the number of offspring reproduced daily by individual *L. trifolii* of age *x* and stage *j* and is determined as $f_{xj}=\frac{E_{xj}}{n_{xj}}$, where *E_xj_* is the sum number of eggs laid by all females at age *x*, and *n_xj_* symbolizes the total number of surviving female adults at age *x*. The age-specific fecundity of the total strain (*m_x_*) represents the mean fecundity of individuals at age *x*, whereas the age-specific maternity (*l_x_m_x_*) is the average number of hatched eggs laid by surviving individuals of age *x*; these are calculated as $m_{x}\frac{\sum_{j=1}^{\beta }s_{xj}f_{xj}}{\sum_{j=1}^{\beta }s_{xj}}$ and $l_{x}m_{x}=l_{x}{*m}_{x}$.

The age-stage life expectancy (*e_xj_*) is the predicted remaining survival time for individuals of age *x* and stage *j* and is calculated as $e_{xj}=\sum_{i=x}^{\infty }\sum_{y=j}^{\beta }{S’}_{iy}$, where ${S’}_{iy}$ is the possibility that an individual of age *x* and stage *j* will survive to age *i* and stage *y* by assuming ${S’}_{iy}$ = 1 [54]. The age-stage reproductive value (*v_xj_*) represents the contribution of individuals of age *x* and stage *j* to the forecasted population and is calculated as $v_{xj}=\frac{e^{r(x+1)}}{s_{xj}}\sum_{i=x}^{\infty }e^{-r(i+1)}\sum_{y=j}^{\beta }{S’}_{iy}f_{iy}$ [55].

The intrinsic rate of increase (*r*) represents the population growth rate as time approaches infinity and the population reaches a stable age-stage distribution, after which the population size will grow at a rate of *e^r^* per time unit, and the finite rate of increase (λ) is the population growth rate as time approaches infinity and the population reaches a stable age-stage distribution, which are calculated as $\sum_{x=0}^{\infty }e^{-r(x+1)}l_{x}m_{x}=1$ and $\lambda =e^{r}$. The net reproductive rate (*R*_0_) is the total average number of offspring that an individual (including females, males, and those that died in the immature stage) can produce and is calculated as $R_{0=}\sum_{x=0}^{\infty }l_{x}m_{x}$. The average generation time (*T*) is the time interval that a population requires to increase its size *R*_0_-fold as time approaches infinity and the population settles down to a stable age-stage distribution, which is calculated as $T=\frac{lnR_{0}}{r}$. *Nf*/*N* represents the percentage of female adults that emerged from the total number of individuals (*N*), and *F* is the mean fecundity of these *Nf* females.

SPSS v. 16.0 software (Chicago, IL, USA, 2008) and one-way analysis of variance (ANOVA) followed by Tukey’s multiple comparison test were used to assess significant differences in eclosion and survival rates, as well as the gene expression of each strain. All data were confirmed to conform to a normal distribution. Student’s *t*-test was employed to compare differences in the average weight of each pupa, preferred temperature, CT_max_ temperature, and gene expression under different treatments. Different uppercase or lowercase letters and asterisks (*) indicate statistically significant differences among treatments at *p* < 0.05.

## 3. Results

### 3.1. Basic Life History Statistics for L. trifolii

#### 3.1.1. Durations of Different Stages and Age-Stage Life Expectancy (*e_xj_*)

The lengths of the egg and larval stages did not differ significantly between the two strains, averaging at approximately 3.5 d for eggs and 3 d for larvae, although male eggs of the AB-R strain did exhibit a significantly prolonged developmental period (3.842 ± 0.086 d) (Table 1). Compared to the pupal development period for the S strain (9.632 ± 0.078 d), selection for abamectin resistance significantly accelerated pupal development (9.283 ± 0.074 d). This acceleration was mainly attributed to the shortened life cycle of male pupae (9.053 ± 0.053 d). Female adults exhibited a longer lifespan than males. In the AB-R strain, the mean lifespans of both female adults (♀: 4.778 ± 0.163 d) and male adults (♂: 3.526 ± 0.141 d) were significantly shorter than those in the S strain (♀: 5.429 ± 0.243 d; ♂: 3.882 ± 0.080 d). Overall, the selection pressure for abamectin resistance in the AB-R strain significantly reduced the mean generation time (*T*) compared to the S strain (Table 2). Female adults of the S strain rarely laid eggs on the first day of emergence (1.048 ± 0.108 d) (Table 1), whereas the AB-R strain displayed slightly earlier egg laying, with an APOP of 0.889 ± 0.061 d. Female adults of the AB-R strain showed a reduced number of days for oviposition (3.889 ± 0.187 d) compared to the S strain (4.381 ± 0.303 d).

**Figure 1:**
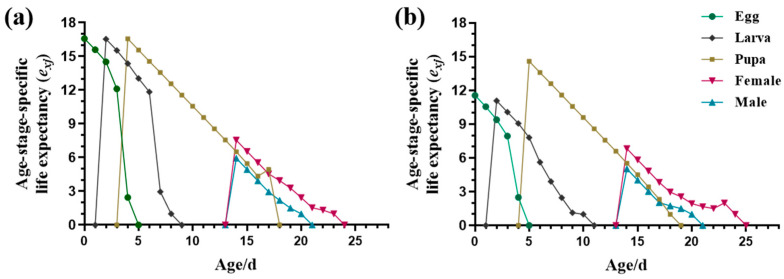
Age-stage-specific life expectancy (*e_xj_*) of different strains of *L. trifolii*. (**a**) Susceptible strain (S strain), (**b**) abamectin-resistant strain (AB-R strain).

As shown in Figure 1, the age-stage life expectancy (*e_xj_*) declined over time for all developmental stages. Initially, the *e_xj_* of a newly laid egg was lower for the AB-R strain compared to the S strain, reaching 11.56 and 16.586 d, respectively. Subsequently, the *e_xj_* of newly hatched larvae and pupae was maintained at approximately 16.5 d for the S strain, while it was lower for AB-R larvae and pupae at 11.075 and 14.586 d, respectively. Moreover, the *e_xj_* of female adults for the S and AB-R strains was 7.553 and 6.833 d, respectively, whereas the newly emerged male adults had *e_xj_* values between 5 and 6 d.

#### 3.1.2. Fecundity and Life Table Parameters

The *r*, *λ*, and *R*_0_ values of the AB-R strain were significantly lower (*r* = 0.108 ± 0.011 d^−1^, *λ* = 1.114 ± 0.012 d^−1^, *R*_0_ = 7.769 ± 1.560 offspring) than those of the S strain (*r* = 0.160 ± 0.012 d^−1^, *λ* = 1.173 ± 0.013 d^−1^, *R*_0_ = 23.483 ± 5.201 offspring) (Table 2).

**Figure 2:**
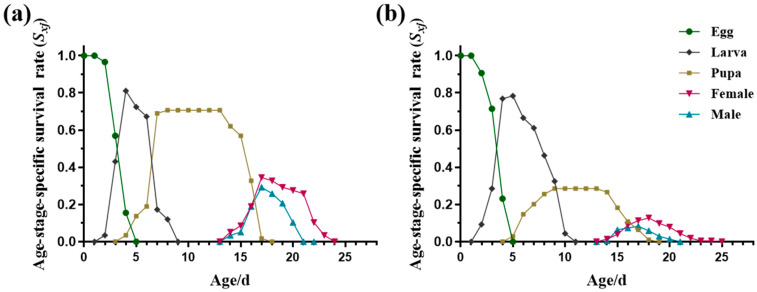
Age-stage-specific survival rates (*s_xj_*) of different strains of *L. trifolii*. (**a**) Susceptible strain (S strain), (**b**) abamectin-resistant strain (AB-R strain).

**Figure 3:**
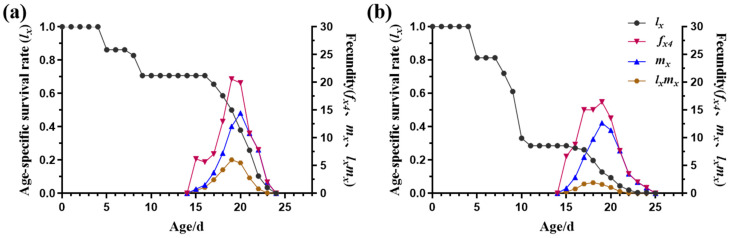
Age-specific survival rates (*l_x_*) and fecundity of different strains of *L. trifolii*. (**a**) Susceptible strain (S strain), (**b**) abamectin-resistant strain (AB-R strain).

Due to variability in developmental rates among individuals, the age-stage-specific survival rates (*s_xj_*) exhibited stage differentiation and significant overlaps in the curves of different stages (Figure 2). For any age (*x*), a newborn could only survive to one of the developmental stages; hence, it was always true that *l_x_* ≤ 1 (Figure 3). For immature *L. trifolii*, the *s_xj_* of the S strain was consistently high, with the highest rate ranging from 70 to 90% (Figure 2a). However, selection for abamectin resistance resulted in a significant decrease in the *s_xj_* of pupae, with values dropping to less than 30% (Figure 2b). The distribution of mortality rates in the larval stage reached 52.71% (Table 3). Therefore, upon exposure to abamectin during the larval stage, the *l_x_* of the AB-R strain showed a consistent and rapid decline until all adult individuals died (Figure 3b). Additionally, the AB-R strain displayed delayed larval development, with some larvae still alive at age 10 d (Figure 2b).

**Figure 4:**
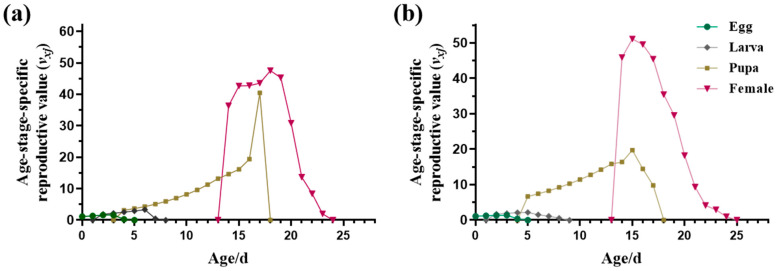
Age-stage-specific reproductive value (*v_xj_*) of different strains of *L. trifolii*. (**a**) Susceptible strain (S strain), (**b**) abamectin-resistant strain (AB-R strain).

*L. trifolii* adults exhibited significantly lower *s_xj_* values compared to the immature stages, and male adults showed lower *s_xj_* values than females. The *s_xj_* of male adults was slightly lower than that of female adults, with the highest *s_xj_* values not exceeding 40% (Figure 2). The impact of insecticide pressure on female adults primarily affected their reproductive capacity. The proportion of females emerging from the total individuals *(Nf*/*N*) in the AB-R strain was significantly lower than in the S strain (Table 2). Consistently, the mean fecundity of females (*F*) was lower in the AB-R strain (58.4074 ± 5.3814 eggs per female) compared to the S strain (64.8571 ± 8.9443 eggs per female) (Table 2). In the AB-R strain, the *f_x4_* and *m_x_* peaks (*f_x_*_4_: 20.59 at age 19 d; *m_x_*: 14.46 at age 20 d) were lower than those of the S strain (*f_x_*_4_: 16.45 at age 19 d; *m_x_*: 12.65 at age 19 d) (Figure 3). Furthermore, *l_x_m_x_* in the AB-R strain peaked at age 18 d with a value of 1.92, significantly lower than in the S strain (6.03 at age 19 d). Female adults were the primary contributors to the future population; *v_xj_* values rapidly increased when the female adults emerged from the pupae, reaching peak values of 47.49 and 51.10 at 18 and 15 d, respectively (Figure 4).

#### 3.1.3. Size of Pupae in Different Strains

**Figure 5:**
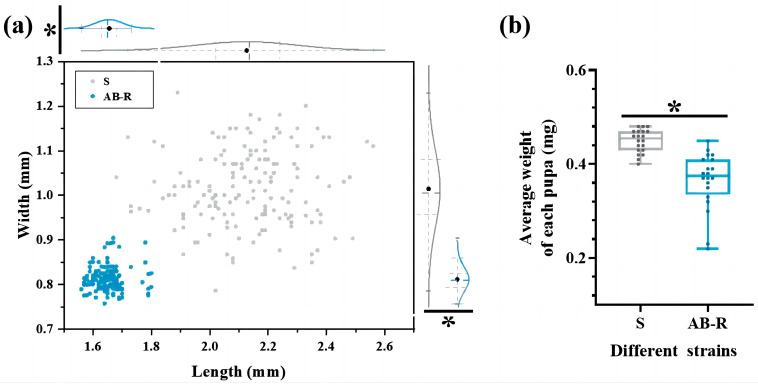
Size of 3-day-old pupae of different strains. (**a**) The length and width of pupae. (**b**) The average weight of each pupa. Student’s *t*-test was used to compare differences between the S and AB-R strains, and the asterisk indicates statistically significant differences between the two strains at *p* < 0.05. Abbreviations: S, susceptible strain; AB-R, abamectin-resistant strain.

Obvious differences in pupal size were evident between the two *L. trifolii* strains (Figure 5a). Pupae of the AB-R strain were significantly smaller than 3-day-old pupae of the S strain, with mean lengths of 2.13 mm and 1.66 mm, respectively (*t* = 32.976, *p* < 0.05), and mean widths of 1.01 mm and 0.81 mm, respectively (*t* = 28.769, *p* < 0.05). The mean weights of pupae showed a correlation with changes in body size (*t* = 5.760, *p* < 0.05) (Figure 5b).

### 3.2. Thermal Preference and Tolerance of L. trifolii

**Figure 6:**
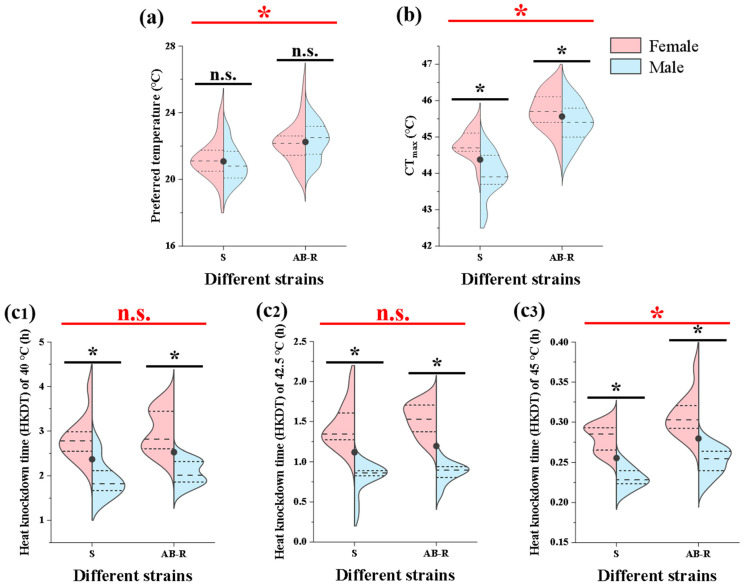
Thermal preference and tolerance of *L. trifolii.* (**a**) The thermal preference of *L. trifolii* adults. (**b**) The critical thermal maximum (CT_max_) temperature of *L. trifolii* adults. (**c**) The heat knockdown time of *L. trifolii* adults in response to different high temperatures. Student’s *t*-test was used to compare differences between the S and AB-R strains. Asterisks indicate statistically significant differences at *p* < 0.05, while n.s. means no significant difference. The black dots represent the average value of each strain. Abbreviations: S, susceptible strain; AB-R, abamectin-resistant strain.

Overall, the T_pref_ of the AB-R strain was predominantly at temperatures of 22 °C and higher, with the highest T_pref_ recorded at 26.0 °C, while the S strain mainly exhibited a T_pref_ of 22 °C or lower, with the lowest T_pref_ reaching 24.4 °C (Figure 6a). The mean T_pref_ of the AB-R strain was 22.128 °C, which was higher than that of the S strain at 21.041 °C (*t* = −6.264, *p* < 0.05). The T_pref_ distribution range was narrower in male adults compared to female adults. In the S strain, the mean T_pref_ of female adults was slightly lower than that of male adults (♀: 21.210, ♂: 20.871; *t* = 1.429, *p* > 0.05), while in the AB-R strain, the opposite trend was observed (♀: 22.206, ♂: 22.050; *t* = 0.618, *p* > 0.05).

Female adults generally displayed more consistent CT_max_ temperatures compared to male adults, with a narrower CT_max_ range and a higher mean CT_max_ temperature (S strain: ♀: 44.8000, ♂: 43.9545; AB-R strain: ♀: 45.7231, ♂: 45.3960) (Figure 6b). Selection for abamectin resistance resulted in a significant increase in the CT_max_ thermal endpoints of *L. trifolii* adults (*t* = −9.609, *p* < 0.05), with the mean CT_max_ rising from 44.3773 °C to 45.5627 °C. Additionally, male adults showed shorter HKDTs and more concentrated HKDT points than females (Figure 6c), with the mean HKDT in the AB-R strain being larger than that in the S strain (Figure 6(c1): *t* = −0.901, *p* > 0.05; Figure 6(c2): *t* = −0.687, *p* > 0.05; Figure 6(c3): *t* = −2.559, *p* > 0.05).

### 3.3. Effect of Thermal Stress on L. trifolii Pupae and Adults

#### 3.3.1. Eclosion Rates of *L. trifolii* Pupae Exposed to Thermal Stress

**Figure 7:**
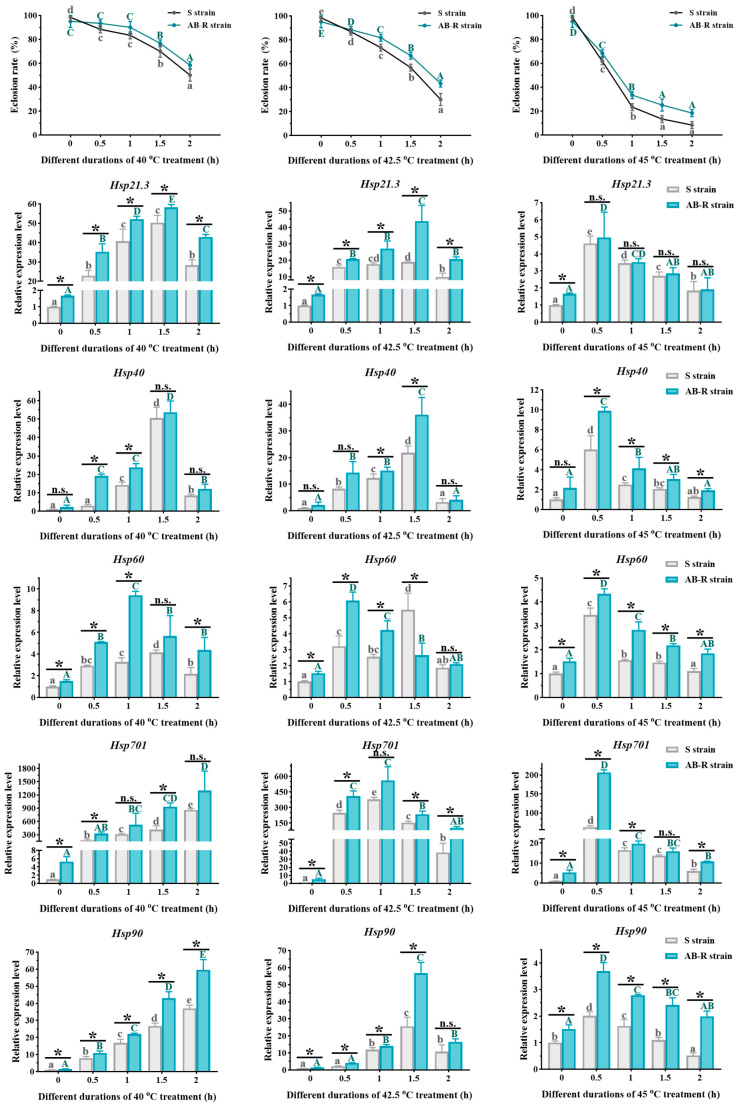
The effect of thermal stress on *L. trifolii* pupae. Susceptible pupae maintained at 25 °C were used as a control group. Data points represent means ± SDs for independent replicates. For ANOVA, data were tested for homogeneity of variances and normality. Different uppercase and lowercase letters indicate significant differences between the different strains. Tukey’s multiple range test was used for the pairwise comparison of means (*p* < 0.05). Asterisks indicate significant differences between the S and AB-R strains, whereas n.s. indicates no significant difference in expression. Abbreviations: S strain, susceptible strain; AB-R strain, abamectin-resistant strain.

As the duration of high-temperature treatment increased, the eclosion rate of *L. trifolii* pupae decreased continuously, with a sharper decline observed at higher treatment temperatures (Figure 7). The AB-R strain exhibited a slightly higher eclosion rate under thermal stress compared to the S strain. Additionally, the disparities between the two strains became more noticeable as the temperature rose and the treatment duration extended.

#### 3.3.2. Survival Rates of *L. trifolii* Adults Exposed to Thermal Stress

**Figure 8:**
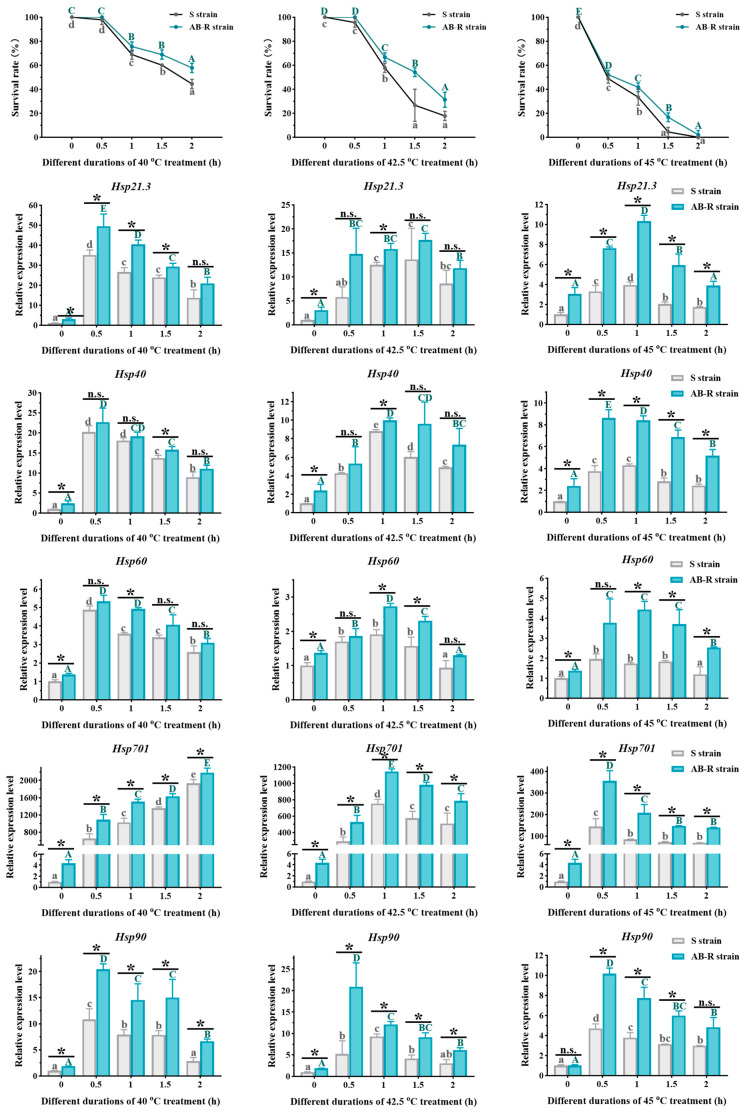
The effect of thermal stress on *L. trifolii* adults. Susceptible adults maintained at 25 °C were used as a control group. Data points represent means ± SDs for independent replicates. For ANOVA, data were tested for homogeneity of variances and normality. Different uppercase and lowercase letters indicate significant differences between the two different strains. Tukey’s multiple range test was used for the pairwise comparison of means (*p* < 0.05). Asterisks indicate significant differences between the S and AB-R strains, whereas n.s. indicates no significant difference in expression. Abbreviations: S strain, susceptible strain; AB-R strain, abamectin-resistant strain.

When compared to the control at 25 °C, the survival rates for both strains exhibited a consistent downward trend as the treatment temperature rose and the duration extended (Figure 8). At 40 °C and 42.5 °C, the survival rates sharply dropped after 1 h of thermal stress. At 45 °C, a significant decline in the survival rate occurred with just 0.5 h of treatment. Overall, the AB-R strain exhibited higher survival rates under thermal stress compared to the S strain. When the treatment temperature reached 45 °C and the duration extended to 1.5 h, almost no S-strain adults survived, while the AB-R strain maintained a survival rate of approximately 20%.

#### 3.3.3. Expression of *LtHsps* in Pupae and Adults Exposed to Thermal Stress

Among the five *LtHsps* in *L. trifolii*, *LtHsp701* showed the highest expression levels under heat stress, while *LtHsp60* was relatively insensitive to elevated temperatures (Figure 7 and Figure 8). The AB-R strain demonstrated higher expression of *LtHsps* compared to the S strain, with the difference being more pronounced under increased thermal stress (45 °C) (Table S2). Overall, *LtHsp* expression was highest at 40 °C, followed by 42.5 °C, and lowest at 45 °C. Additionally, the higher the treatment temperature, the earlier the peak expression occurred. At 40 °C, the highest expression of *LtHsp21.3*, *LtHsp40*, *LtHsp60*, *LtHsp701*, and *LtHsp90* was observed at 1.5 h, 1.5 h, 1 h, 2 h, and 2 h, respectively; at 42.5 °C, the highest expression was observed at 1.5 h, 1.5 h, 0.5 h, 1 h, and 1.5 h for the mentioned *LtHsps*. When the temperature reached 45 °C, peak expression for all *LtHsps* occurred at 0.5 h, followed by a continuous decline as the treatment duration extended.

## 4. Discussion

There is substantial evidence indicating that insecticide resistance results in both fitness costs and benefits in pests, while the impact of insecticide resistance on the thermotolerance of pests is mostly unclear [56,57]. This study aimed to explore the potential adaptive response of *L. trifolii* to abiotic stress resistance, specifically heat shock caused by the development of insecticide resistance. Employing an abamectin-resistant strain, we examined the effects of selection pressure for insecticide resistance on the population dynamics of *L. trifolii* through a comprehensive evaluation of life history parameters. Furthermore, we assessed the altered ability to withstand extreme high-temperature stress caused by enhanced insecticide resistance.

Although the total longevity of the AB-R strain was comparable to that of the S strain (Table 1), long-term selection for abamectin resistance resulted in delayed larval development and approximately 50% mortality in the AB-R strain (Figure 1, Table 3). Surviving pupae exhibited smaller body sizes and accelerated development (Figure 5). These changes could be caused by a reallocation of resources in the AB-R larvae toward prioritizing detoxification and metabolic processes in the gut, which could impact nutrient absorption, digestion, and conversion efficiency [9,58]. Alternatively, insects may have been attempting to minimize their exposure to abamectin to prevent poisoning. This demonstrates that the effect of chemical insecticides on pests is dual in nature. Although insecticides can effectively eliminate most pests, they can also trigger the development of self-defense mechanisms in insects, ultimately resulting in the rapid adaptation of the population.

The reproductive and survival abilities of *L. trifolii* adults play critical roles in the dissemination and multiplication of populations [16]. Abamectin had long-lasting effects on *L. trifolii* from immature to mature stages, leading to a significant decrease in *sxj* and all fecundity parameters. This suggests the prolonged efficacy of abamectin throughout the insect’s life cycle (Figure 2 and Figure 3, Table 2). The adult lifespan of the AB-R strain was significantly shorter than that of the S strain, with a significant decrease in all reproductive indicators observed in the AB-R strain (Figure 1). Additionally, female adults generally did not lay eggs on the first day of emergence, consistent with previous findings [59]; however, the AB-R strain showed slightly earlier oviposition (Table 1).

Differences in thermal preference and tolerance were observed between the two strains, with the AB-R strain showing a higher thermal preference and stronger tolerance to adverse temperatures than the S strain. The AB-R strain preferred higher temperatures (22 °C and above) compared to the S strain (22 °C or below) and exhibited higher CT_max_ temperatures and longer HKDTs (Figure 6). Under thermal stress, the AB-R strain maintained higher eclosion and survival rates than the S strain, particularly at 45 °C, where the AB-R strain showed about 20% survival at 1.5 h, while almost no individuals in the S strain survived (Figure 7 and Figure 8). Additionally, the expression of *LtHsps* was found to be highest at 40 °C, followed by 42.5 °C, and lowest at 45 °C, with peak expression occurring earlier at higher temperatures. The earlier peak expression of *LtHsps* at higher temperatures indicated a rapid response mechanism to thermal stress, consistent with the adaptive plasticity observed in other studies [24,25]. The AB-R strain exhibited higher *LtHsp* expression to mitigate thermal damage compared to the S strain, with the difference being more pronounced under increased thermal stress (45 °C). The production of HSPs in *Tetranychus cinnabarinus* (Boisduval) and *Bactrocera cucurbitae* (Coquillett) was higher in the abamectin-resistant strain compared to the susceptible strain, suggesting that adaptation to thermal environments might assist organisms in overcoming insecticide toxicity by inducing *Hsp* expression to resist oxidative damage [60,61]. These results suggest that the increased selection pressure for abamectin resistance can induce the development of thermotolerance, and the potential adaptive cross-tolerance between thermal and insecticidal stresses should be taken into consideration when developing effective pest management strategies in the context of global warming.

## 5. Conclusions

Our results demonstrate that selecting for abamectin resistance during the larval stage of *L. trifolii*, though detrimental to the population’s survival and reproduction, also activated self-defense mechanisms and rapid adaptive adjustments and led to a modest increase in thermal tolerance. The enhanced insecticide tolerance of *L. trifolii* resulted in higher thermal preference, elevated CT_max_ values, longer HKDTs, improved survival, and the upregulation of *LtHsps* during heat stress. This indicates that the evolution of thermal adaptation was accompanied by the development of abamectin resistance, emphasizing the importance of considering temperature in the application of chemical control for *L. trifolii* amidst global climate change. Our study provides valuable insights into how physiological acclimation may help mitigate the toxic effects of insecticides and illustrates how insects respond to multiple environmental pressures.

## Figures and Tables

**Table 1:** Mean durations of different stages of *L. trifolii*. The ‘Total’ rows include all individuals, including those that did not survive to complete the entire developmental cycle. The ‘Female’ and ‘Male’ rows represent the mean durations for individuals that successfully completed the entire developmental cycle. Mean durations are presented with standard deviations (Mean ± SD). Student’s *t*-test was used to compare differences between the S and AB-R strains, and different lowercase letters indicate statistically significant differences between the two strains at *p* < 0.05. Abbreviations: APOP, adult pre-oviposition period of female adult; TPOP, total pre-oviposition period of female counted from birth; Ovi-days, oviposition days, the mean number of days that an insect has laid eggs.

Developmental Stages		Strains		
		S	AB-R	*p* Value
Egg (d)	Total	3.480 ± 0.086	3.588 ± 0.060	0.3062
	Female	3.333 ± 0.125	3.482 ± 0.098	0.35522
	Male	3.412 ± 0.172 b	3.842 ± 0.086 a	0.02681
Larva (d)	Total	3.098 ± 0.119	3.069 ± 0.140	0.87704
	Female	3.191 ± 0.189	3.148 ± 0.197	0.87845
	Male	3.000 ± 0.171	2.684 ± 0.242	0.28939
Pupa (d)	Total	9.632 ± 0.078 a	9.283 ± 0.074 b	0.00111
	Female	9.619 ± 0.107	9.444 ± 0.111	0.26098
	Male	9.647 ± 0.118 a	9.053 ± 0.053 b	0.00006
Adult (d)	Total	4.737 ± 0.186 a	4.261 ± 0.144 b	0.04344
	Female	5.429 ± 0.243 a	4.778 ± 0.163 b	0.02657
	Male	3.882 ± 0.080 a	3.526 ± 0.141 b	0.02881
APOP (d)		1.048 ± 0.108	0.889 ± 0.061	0.2086
TPOP (d)		17.191 ± 0.296	16.963 ± 0.0.222	0.53915
Ovi-days		4.381 ± 0.303	3.889 ± 0.187	0.16704
Total longevity		20.842 ± 0.271	20.130 ± 0.246	0.05013

**Table 2:** Life table parameters of different strains of *L. trifolii*. Mean parameters are presented with standard deviations (Mean ± SD). Student’s *t*-test was used to compare differences between the S and AB-R strains, and different lowercase letters indicate statistically significant differences between the two strains at *p* < 0.05. Abbreviations: *r*, the intrinsic rate of increase, the population growth rate as time approaches infinity and the population reaches a stable age-stage distribution, after which the population size will increase at a rate of er per time unit; *λ*, the finite rate of increase, the population growth rate as time approaches infinity and the population reaches a stable age-stage distribution; *R*_0_, the net reproductive rate, the total mean number of offspring that an average individual (including females, males, and those that died in the immature stage) can produce during its lifetime; *T*, the mean generation time, the period that a population requires to increase its size *R*_0_-fold as time approaches infinity and the population settles down to a stable age-stage distribution; *Nf*/*N*, the proportion of female adults emerged from the total individuals *N*; *F*, fecundity, the mean fecundity of these *Nf* females.

Population Parameters	Strains		
	S	AB-R	*p* Value
*r* (day^−1^)	0.160 ± 0.012 a	0.108 ± 0.011 b	0.00231
*λ* (day^−1^)	1.173 ± 0.013 a	1.114 ± 0.012 b	0.00209
*R*_0_ (offspring/individual)	23.483 ± 5.201 a	7.769 ± 1.560 b	0.00424
*T* (days)	19.771 ± 0.267 a	19.019 ± 0.244 b	0.03941
*Nf*/*N*	0.362 ± 0.063 a	0.133 ± 0.024 b	0.00077
*F* (eggs per female)	64.857 ± 8.944	58.407 ± 5.381	0.53589

**Table 3:** Distribution of mortality rates of *L. trifolii* in each stage. Mean values are presented with standard deviations (Mean ± SD). Student’s *t*-test was used to compare differences between the S and AB-R strains, and different lowercase letters indicate statistically significant differences between the two strains at *p* < 0.05.

Developmental Stages		Strains		
		S	AB-R	*p* Value
Immature stage	Egg	0.138 ± 0.045	0.187 ± 0.027	0.36342
	Larva	0.155 ± 0.048 b	0.527 ± 0.035 a	*p* < 0.00001
	Pupa	0.052 ± 0.029	0.059 ± 0.017	0.85398
Mature stage	Female	0.362 ± 0.063 a	0.133 ± 0.024 b	0.00077
	Male	0.239 ± 0.060 a	0.094 ± 0.021 b	0.00178

## Data Availability

Data are contained within the article or Supplementary Materials. The original contributions presented in the study are included in the article/Supplementary Materials. Further inquiries can be directed to the corresponding author/s.

## Supplementary Materials

The following supporting information can be downloaded at https://www.mdpi.com/article/10.3390/insects15060462/s1: Table S1: Primers used for real-time quantitative PCR.; Table S2: Significant differences of Student’s *t*-test and ANOVA.
