# Supplementary material for: Life Table Study of Liriomyza trifolii and Its Contribution to Thermotolerance: Responding to Long-Term Selection Pressure for Abamectin Resistance
Source: Insects. 2024 Jun 20;15(6):462. doi: 10.3390/insects15060462 (PMC11203713; doi:10.3390/insects15060462)
Supplement: Supplementary file 1 [file insects-15-00462-s001.zip › insects-3043739-supplementary.pdf]

Supplementary materials

Table S1. Primers used for real-time quantitative PCR. Abbreviations: F, forward; R, reverse.

Gene	GenBank Accession no.		Primer Sequences	Fragment Length (bp)
Hsp21.3	KY231145	F	GAAATCAATGTGAAAGTGGTGGA	175
		R	GAACCTTCAACAAGCCATCAGAT	
Hsp701	KY933451	F	CAAATCCCAGAGCCTTGAGAC	173
		R	GCTACGGAATAAGTCAGCACAAA	
Hsp40	KY231146	F	AAAGTCTCACTCAAGCAAGCATT	127
		R	GTCCAGTTATGCGTTTGACAGTT	
Hsp60	KY231147	F	AAATAGTGCGTCGTTCATTGCGT	99
		R	CGGATTGTGTTTCAACTTTAGCC	
Hsp90	KY231149	F	CAAAACTAAACCCATCTGGACACG	158
		R	GCACAAACAAAAGAGCACGGA	
Actin	KY231150	F	TTGTATTGGACTCTGGTGACGG	73
		R	GATAGCGTGAGGCAAAGCATAA	

Table S2. Significant differences of Student's *t*-test and ANOVA. Abbreviations: S, susceptible strain; AB-R, abamectin-resistant strain.

Temperatures (°C)		Strains		Durations (h)				
		S	AB-R	0	0.5	1	1.5	2
**Pupa**								
40	Eclosion rate	$F_{4,10}=69.833$, $p < 0.05$	$F_{4,10}=47.389$, $p < 0.05$					
	Hsp21.3	$F_{4,11}=67.082$, $p < 0.05$	$F_{4,10}=314.920$, $p < 0.05$	$t = -18.861$, $p < 0.05$	$t = -4.315$, $p < 0.05$	$t = -3.021$, $p < 0.05$	$t = -3.331$, $p < 0.05$	$t = -8.072$, $p < 0.05$
	Hsp40	$F_{4,12}=182.954$, $p < 0.05$	$F_{4,12}=111.946$, $p < 0.05$	$t = -1.847$, $p > 0.05$	$t = -22.553$, $p < 0.05$	$t = -7.988$, $p < 0.05$	$t = -0.664$, $p > 0.05$	$t = -2.350$, $p > 0.05$
	Hsp60	$F_{4,12}=38.707$, $p < 0.05$	$F_{4,12}=35.854$, $p < 0.05$	$t = -6.396$, $p < 0.05$	$t = -57.736$, $p < 0.05$	$t = -22.455$, $p < 0.05$	$t = -1.359$, $p > 0.05$	$t = -2.953$, $p < 0.05$
	Hsp701	$F_{4,13}=255.178$, $p < 0.05$	$F_{4,11}=13.975$, $p < 0.05$	$t = -6.038$, $p < 0.05$	$t = -6.136$, $p < 0.05$	$t = -1.607$, $p > 0.05$	$t = -7.502$, $p < 0.05$	$t = -1.717$, $p > 0.05$
	Hsp90	$F_{4,13}=274.157$, $p < 0.05$	$F_{4,12}=197.576$, $p < 0.05$	$t = -5.987$, $p < 0.05$	$t = -3.305$, $p < 0.05$	$t = -5.229$, $p < 0.05$	$t = -6.770$, $p < 0.05$	$t = -6.297$, $p < 0.05$
42.5	Eclosion rate	$F_{4,10}=184.143$, $p < 0.05$	$F_{4,10}=108.929$, $p < 0.05$					
	Hsp21.3	$F_{4,11}=125.175$, $p < 0.05$	$F_{4,11}=30.209$, $p < 0.05$	$t = -18.861$, $p < 0.05$	$t = -6.434$, $p < 0.05$	$t = -4.021$, $p < 0.05$	$t = -4.480$, $p < 0.05$	$t = -7.129$, $p < 0.05$
	Hsp40	$F_{4,12}=95.243$, $p < 0.05$	$F_{4,12}=49.527$, $p < 0.05$	$t = -1.847$, $p > 0.05$	$t = -2.466$, $p > 0.05$	$t = -3.056$, $p < 0.05$	$t = -3.583$, $p < 0.05$	$t = -0.744$, $p > 0.05$
	Hsp60	$F_{4,11}=30.354$, $p < 0.05$	$F_{4,14}=53.889$, $p < 0.05$	$t = -6.396$, $p < 0.05$	$t = -5.881$, $p < 0.05$	$t = -5.495$, $p < 0.05$	$t = 3.806$, $p < 0.05$	$t = -1.953$, $p > 0.05$
	Hsp701	$F_{4,12}=290.122$, $p < 0.05$	$F_{4,12}=41.482$, $p < 0.05$	$t = -6.038$, $p < 0.05$	$t = -6.160$, $p < 0.05$	$t = -2.389$, $p > 0.05$	$t = -4.224$, $p < 0.05$	$t = -6.533$, $p < 0.05$
	Hsp90	$F_{4,12}=40.498$, $p < 0.05$	$F_{4,12}=200.526$, $p < 0.05$	$t = -5.987$, $p < 0.05$	$t = -9.656$, $p < 0.05$	$t = -3.208$, $p < 0.05$	$t = -6.485$, $p < 0.05$	$t = -2.331$, $p > 0.05$

	Eclosion rate	$F_{4,10}$ = 527.300, $p < 0.05$	$F_{4,10}$ = 212.333, $p < 0.05$					
	Hsp21.3	$F_{4,12}$ = 61.302, $p < 0.05$	$F_{4,12}$ = 11.319, $p < 0.05$	$t = −18.861$, $p < 0.05$	$t = −0.396$, $p > 0.05$	$t = −0.481$, $p > 0.05$	$t = −0.598$, $p > 0.05$	$t = −0.141$, $p > 0.05$
	Hsp40	$F_{4,12}$ = 35.265, $p < 0.05$	$F_{4,11}$ = 52.421, $p < 0.05$	$t = −1.847$, $p > 0.05$	$t = −4.620$, $p < 0.05$	$t = −3.332$, $p < 0.05$	$t = −3.392$, $p < 0.05$	$t = −6.025$, $p < 0.05$
45	Hsp60	$F_{4,11}$ = 158.811, $p < 0.05$	$F_{4,14}$ = 86.905, $p < 0.05$	$t = −6.396$, $p < 0.05$	$t = −4.230$, $p < 0.05$	$t = −8.362$, $p < 0.05$	$t = −13.318$, $p < 0.05$	$t = −6.041$, $p < 0.05$
	Hsp701	$F_{4,13}$ = 611.424, $p < 0.05$	$F_{4,12}$ = 241.58, $p < 0.05$	$t = −6.038$, $p < 0.05$	$t = −30.778$, $p < 0.05$	$t = −3.965$, $p < 0.05$	$t = −2.292$, $p > 0.05$	$t = −9.506$, $p < 0.05$
	Hsp90	$F_{4,11}$ = 52.859, $p < 0.05$	$F_{4,10}$ = 41.859, $p < 0.05$	$t = −5.987$, $p < 0.05$	$t = −8.184$, $p < 0.05$	$t = −8.145$, $p < 0.05$	$t = −7.851$, $p < 0.05$	$t = −11.387$, $p < 0.05$
**Adult**								
	Survival rate	$F_{4,10}$ = 197.167, $p < 0.05$	$F_{4,10}$ = 121.167, $p < 0.05$					
	Hsp21.3	$F_{4,10}$ = 92.021, $p < 0.05$	$F_{4,13}$ = 133.009, $p < 0.05$	$t = −6.420$, $p < 0.05$	$t = −3.822$, $p < 0.05$	$t = −8.673$, $p < 0.05$	$t = −4.588$, $p < 0.05$	$t = −2.504$, $p > 0.05$
	Hsp40	$F_{4,10}$ = 179.376, $p < 0.05$	$F_{4,12}$ = 83.979, $p < 0.05$	$t = −3.527$, $p < 0.05$	$t = −1.106$, $p > 0.05$	$t = −1.402$, $p > 0.05$	$t = −3.507$, $p < 0.05$	$t = −2.326$, $p > 0.05$
40	Hsp60	$F_{4,11}$ = 181.423, $p < 0.05$	$F_{4,12}$ = 120.890, $p < 0.05$	$t = −6.644$, $p < 0.05$	$t = −2.057$, $p > 0.05$	$t = −29.385$, $p < 0.05$	$t = −2.149$, $p > 0.05$	$t = −2.117$, $p > 0.05$
	Hsp701	$F_{4,12}$ = 238.915, $p < 0.05$	$F_{4,11}$ = 312.780, $p < 0.05$	$t = −8.789$, $p < 0.05$	$t = −4.559$, $p < 0.05$	$t = −8.724$, $p < 0.05$	$t = −7.049$, $p < 0.05$	$t = −3.052$, $p < 0.05$
	Hsp90	$F_{4,11}$ = 48.134, $p < 0.05$	$F_{4,12}$ = 40.866, $p < 0.05$	$t = −18.670$, $p < 0.05$	$t = −7.176$, $p < 0.05$	$t = −3.531$, $p < 0.05$	$t = −3.466$, $p < 0.05$	$t = −7.954$, $p < 0.05$
	Survival rate	$F_{4,10}$ = 97.233, $p < 0.05$	$F_{4,10}$ = 205.200, $p < 0.05$					
42.5	Hsp21.3	$F_{4,11}$ = 8.386, p	$F_{4,12}$ = 20.417, $p <$	$t = −6.420$,	$t = −2.676$,	$t = −5.002$,	$t = −1.052$,	$t = −1.717$,

	< 0.05	0.05	$p < 0.05$	$p > 0.05$	$p < 0.05$	$p > 0.05$	$p > 0.05$
Hsp40	$F_{4,10} = 327.154$, $p < 0.05$	$F_{4,12} = 17.601$, $p < 0.05$	$t = -3.527$, $p < 0.05$	$t = -1.004$, $p > 0.05$	$t = -7.368$, $p < 0.05$	$t = -2.549$, $p > 0.05$	$t = -1.850$, $p > 0.05$
Hsp60	$F_{4,12} = 22.597$, $p < 0.05$	$F_{4,12} = 98.748$, $p < 0.05$	$t = -6.644$, $p < 0.05$	$t = -1.054$, $p > 0.05$	$t = -10.116$, $p < 0.05$	$t = -4.466$, $p < 0.05$	$t = -2.928$, $p > 0.05$
Hsp701	$F_{4,10} = 43.843$, $p < 0.05$	$F_{4,11} = 201.129$, $p < 0.05$	$t = -8.789$, $p < 0.05$	$t = -4.131$, $p < 0.05$	$t = -12.530$, $p < 0.05$	$t = -7.744$, $p < 0.05$	$t = -3.068$, $p < 0.05$
Hsp90	$F_{4,12} = 20.136$, $p < 0.05$	$F_{4,12} = 31.014$, $p < 0.05$	$t = -18.670$, $p < 0.05$	$t = -4.280$, $p < 0.05$	$t = -6.095$, $p < 0.05$	$t = -6.489$, $p < 0.05$	$t = -5.230$, $p < 0.05$
Survival rate	$F_{4,10} = 331.700$, $p < 0.05$	$F_{4,10} = 410.375$, $p < 0.05$					
Hsp21.3	$F_{4,11} = 46.287$, $p < 0.05$	$F_{4,12} = 76.007$, $p < 0.05$	$t = -6.420$, $p < 0.05$	$t = -12.166$, $p < 0.05$	$t = -19.335$, $p < 0.05$	$t = -5.965$, $p < 0.05$	$t = -8.310$, $p < 0.05$
Hsp40	$F_{4,11} = 75.266$, $p < 0.05$	$F_{4,12} = 66.006$, $p < 0.05$	$t = -3.527$, $p < 0.05$	$t = -9.236$, $p < 0.05$	$t = -18.740$, $p < 0.05$	$t = -9.594$, $p < 0.05$	$t = -8.320$, $p < 0.05$
Hsp60	$F_{4,11} = 13.478$, $p < 0.05$	$F_{4,11} = 13.501$, $p < 0.05$	$t = -6.644$, $p < 0.05$	$t = -2.583$, $p > 0.05$	$t = -11.550$, $p < 0.05$	$t = -4.452$, $p < 0.05$	$t = -6.153$, $p < 0.05$
Hsp701	$F_{4,12} = 26.750$, $p < 0.05$	$F_{4,11} = 58.893$, $p < 0.05$	$t = -8.789$, $p < 0.05$	$t = -6.684$, $p < 0.05$	$t = -6.296$, $p < 0.05$	$t = -41.726$, $p < 0.05$	$t = -56.024$, $p < 0.05$
Hsp90	$F_{4,12} = 79.982$, $p < 0.05$	$F_{4,12} = 78.336$, $p < 0.05$	$t = -18.670$, $p < 0.05$	$t = -13.094$, $p < 0.05$	$t = -5.777$, $p < 0.05$	$t = -10.902$, $p < 0.05$	$t = -3.281$, $p > 0.05$
